# Trusting COVID-19 vaccines as individual and social goal

**DOI:** 10.1038/s41598-022-13675-3

**Published:** 2022-06-08

**Authors:** Rino Falcone, Alessandro Ansani, Elisa Colì, Marco Marini, Alessandro Sapienza, Cristiano Castelfranchi, Fabio Paglieri

**Affiliations:** 1 Trust Theory and Technology Group, Institute of Cognitive Sciences and Technologies, National Research Council of Italy, Rome, Italy; 2 Goal-Oriented Agents Lab, Institute of Cognitive Sciences and Technologies, National Research Council of Italy, Rome, Italy; 3 Department of Psychology, Sapienza University of Rome, Rome, Italy; 4 CoSMIC Lab, Department of Philosophy, Communication and Performing Arts, Roma Tre University, Rome, Italy; 5 Evaluation Research Group, Institute of Cognitive Sciences and Technologies, National Research Council of Italy, Rome, Italy

**Keywords:** Psychology, Human behaviour

## Abstract

Trust in vaccines and in the institutions responsible for their management is a key asset in the global response to the COVID-19 pandemic. By means of a structured multi-scales survey based on the socio-cognitive model of trust, this study investigates the interplay of institutional trust, confidence in COVID-19 vaccines, information habits, personal motivations, and background beliefs on the pandemic in determining willingness to vaccinate in a sample of Italian respondents (N = 4096). We observe substantial trust in public institutions and a strong vaccination intention. Theory-driven structural equation analysis revealed what factors act as important predictors of willingness to vaccinate: trust in vaccine manufacturers (which in turn is supported by trust in regulators), collectivist goals, self-perceived knowledgeability, reliance on traditional media for information gathering, and trust in institutional and scientific sources. In contrast, vaccine hesitancy, while confined to a minority, is more prominent in less educated and less affluent respondents. These findings can inform institutional decisions on vaccine communication and vaccination campaigns.

## Introduction

Defeating the COVID-19 pandemic constitutes an unprecedented challenge for societies all over the world. Among other things, it pressures us to rethink our entire framework of relationships and the most basic collaborative dynamics, both at the individual level and across all social organizations (local and global; public and private; general and specialized). Faced with the need for effective cooperation on a global scale under conditions of deep uncertainty, it is not surprising that trust is quickly emerging as a key commodity for behavioural interventions against COVID-19^1,2^: not only trust in each other, but also in public authorities, scientific experts, and even the private sector.

The uneven success of COVID-19 vaccine rollout across the globe provides an ideal example of the pivotal role of trust in pandemic response. The particularly rapid development of safe and effective vaccines against COVID, achieved thanks to an extraordinary mobilization of advanced research, scientific interaction between laboratories of excellence, and public and private capital, marked a landmark result in medicine and the first step in bringing the world back to a normal situation. However, the next stage in our fight against the pandemic is proving no less challenging: inoculating COVID-19 vaccines to a sufficiently large portion of the world population, in order to definitely curtail the spread of the virus, entails momentous logistical and financial efforts, which are currently still far from being completed: as of April 13, 2022, more than 11 billions of doses have been administered worldwide, but only 4.6 billions of people are fully vaccinated (59%, as compared to the 5.7% at the time of data collection^3^), that is still far from the WHO goal of 70% for the mid-2022 (see https://www.who.int/news/item/23-12-2021-achieving-70-covid-19-immunization-coverage-by-mid-2022).

What is worst, enormous inequalities emerged in vaccine rollout, often mirroring limited access to vaccines due to financial constraints (e.g., in many countries in Africa^4^), excessive caution in granting approval of foreign-produced drugs (e.g., in Japan^5^), or supply problems (e.g., in Australia^4^).

In light of the logistical complexities involved in producing, distributing, and administering vaccines on such a global scale, widespread acceptance of these drugs and a robust intention to vaccinate as soon as possible become essential to success. This is where trust in vaccines manifests its centrality: not only trust in their effectiveness and safety, but also trust in the authorities that monitor their development and their quality, as well as in the companies that produce them. An attitude of trust that has to emerge in the context of vaccination campaigns that, across the whole world, are currently mobilizing extraordinary human and financial resources, with all the additional complexities entailed by such huge interests. If such an attitude is not properly nurtured and scaffolded in the population, we are at risk of stumbling on the last mile in our collective response to the pandemic^6^.

Even though the key link between trust and vaccination is fully acknowledged by relevant authorities, e.g. the WHO^7^, there are still too few studies dedicated to explore what factors affect such trust and how it translates to intention to vaccinate^8^: this is a regrettable oversight, since recent evidence shows the key role of trust in promoting other pro-social behaviours needed to contain the pandemic^9^. Therefore, in order to fill this research gap, we have investigated not only the role of trust in its different shapes but also its causes and reasons, observing the complex causal model behind the decision to vaccinate. The use of this approach represents an added value, which is possible thanks to the use of a theoretical model already structured in itself^10^. Indeed, the validity of causal connections and our theoretical hypotheses were further verified through SEM models that allowed us to clearly outline the path between trust, source of information and self-awareness, and the willingness to vaccinate. To investigate how trust dynamics may play out for COVID-19 vaccines and affect vaccination intentions, we decided to use Italy as a case study. This country provides an interesting testbed for assessing the role of trust in modulating vaccination behaviour, since it used to combine, prior to the onset of the pandemic, very low levels of trust towards public institutions with relatively low levels of vaccine hesitancy. Italians historically do not trust easily their political authorities^11,12^, yet they tend to be willing to vaccinate (much more, for instance, than French people, in spite of many similarities between these two countries^13^). Thus any interplay between institutional trust and willingness to vaccinate in an Italian sample is likely to reveal interesting underlying dynamics, rather than a simple tendency to comply with the indications of the relevant authorities.

The theoretical model we used to structure our investigation^10,14^ considers trust as a complex socio-cognitive phenomenon: a state and mental attitude of a hybrid nature (both affective and cognitive), with a composite structure (made of different ingredients: beliefs, purposes, intentions, expectations, etc.), oriented to different entities and dimensions, and inherently recursive and dynamic (not only because it changes over time, but also because trust can derive from trust: reciprocity, transitivity, categorization, etc.).

It is worth noting that vaccination against COVID-19 started in Italy since the beginning of January 2021. The vaccines in use at the time of data collection, purchased by the European Union and distributed to member countries including Italy, were four: Pfizer, Moderna, AstraZeneca (now Vaxzevria), and Johnson & Johnson (the latter not yet in use at the time of the survey, since the authorization of the EMA was still pending). The first two are m-RNA based, whereas the others use a viral vector.

The main objective of this investigation was to characterize whether, how and to what extent respondents would trust COVID-19 vaccines, as well as the relevant policies for their deployment and the main communication channels used to inform about them, and how these attitudes would affect their intention to vaccinate. At the time when this survey was conducted, the scenario was still quite dire for the Italian population: the country was in the grip of the third wave of the pandemic, the death toll had surpassed one hundred thousand people in Italy alone, the vaccination campaign was struggling to reach its intended rollout objectives, exceedingly restrictive social constraints were still being enforced, large sectors of the economy were in severe crisis (like in so many other countries across the world), and the AstraZeneca PR fiasco^15^ had just transpired in Europe, fueling the flames of vaccine hesitancy at the least appropriate moment.

Against the backdrop of this scenario, we intended to address 6 main areas of interest (AoI):

1. How is trust in institutions evolving in relation to the pandemic, after more than one year of this global emergency? Given the importance of trust in national governments to support vaccination campaigns^16^, what is the public perception of these public institutions?
2. To what extent citizens place their trust in different actors involved in vaccination campaigns? Is trust in, respectively, regulators and manufacturers based on different reasons? How these different types of trust interact with each other, and how do they influence intention to vaccinate?
3. What kind of background beliefs on vaccination do people harbor, and how do these attitudes modulate their response to COVID-19 vaccines? How is vaccine hesitancy linked to this unprecedented global health crisis?
4. Do citizens consider themselves adequately informed about COVID-19 vaccines, and how does this impact their willingness to vaccinate? What channels do they use more often, and what information sources do they typically trust? How do these information habits affect intention to vaccinate, against the potential interference of misinformation campaigns^17^ and anti-intellectualist sentiment^18^?
5. What are the main motives supporting people’s intention to vaccinate? Are some of these reasons more effective than others in promoting vaccination?
6. What socio-demographic factors are more relevant to determine willingness to vaccinate? In particular, what is the role of prior education and income level in modulating vaccination beliefs and intentions?

Answering these questions will help us better understand how trust can improve our collective response to the ongoing pandemic, especially in relation to the need to ensure rapid and widespread administration of COVID-19 vaccines worldwide. To what extent such response will prove to be exportable to other domains where collective action is equally urgent, such as sustainable development, socioeconomic equity, and cultural integration, remains to be seen: yet there is hope that the current plight we are facing together will allow us to learn valuable lessons to increase global preparedness.

## Results

### Sample characteristics

The sample analyzed included 4096 people, interviewed between March 26 and April 7. All participants were Italian native speakers between 18 and 87 years old (mean age = 49 years, *SD* = 12.59). Concerning the geographical distribution, 36% came from Northern Italy (NI), 37% from Central Italy (CI), and 27% from Southern Italy and main islands (SI). In terms of educational background, 26% had a high school diploma or lower title, 36% were University graduates, 38% had completed a post-graduate specialization.

Before running the analysis, we made sure that our sample could be comparable to the current Italian population in terms of vaccination (23.4%, at the date of data collection), mean age, gender, and geographical distribution (the data of the Italian population were 24.4%, 46.2%, F = 51.3%, NI = 41%, CI 32%, SI = 26% respectively, as reported by www.istat.it). See Table 1 for a detailed description of our sample.

**Table 1:** Sample characteristics.

	Male % (44%)	Female % (56%)	Total %
**Age (mean = 46)**			
18–29	9	7	8
30–39	14	16	15
40–49	24	29	28
50–59	26	30	28
60–69	18	15	17
> 70	6	3	4
Total	100	100	100
**Educational background**			
High school or lower	25	27	26
University degree	37	35	36
Post-graduate specialization	38	38	38
Total	100	100	100
**Geographical distribution**			
Northern Italy	37	34	36
Central Italy	36	39	37
Southern Italy/islands	27	27	27
Total	100	100	100

### Survey responses

The single, most striking finding of this survey is the high level of *trust in public authorities* manifested by participants, with respect to the management of the pandemic: 75.7% of respondents expressed either high (24.8%) or good (50.9%) confidence in the institutions’ ability to deal with this crisis, in collaboration (explicit synergy) with the competent medical authorities. However, a significant erosion in that trust was still apparent. The current survey was designed to allow comparison with the data on institutional trust collected by Falcone and collaborators in March 2020 (with a different population of 4260 participants, but similar socio-demographic characteristics, comparable methods, repeated items, and based on the same theoretical framework; see^14^): this revealed both an increase in explicit distrust towards institutions (from 7.3% in 2020 to 24.3% now) and a comparable reduction in those who expressed complete confidence in the choices made by public authorities (from 23.8 to 2.9%)—AoI 1 in the introduction.

Importantly, participants’ institutional trust has a rather specific target: when asked to indicate what is the most relevant institution for dealing with the pandemic, among the *various competent authorities* (national, regional, municipal, etc.), the vast majority of respondents (74.5%) opted for the central national government. This seems to reflect an appreciation of the part played by the central government and by other national authorities (Civil Protection, 9.1%, and the Presidency of the Republic, 4.9%) in orchestrating the Italian response to the pandemic, whereas regional (7.1%) and municipal (2.2%) authorities are mostly perceived as having an ancillary role, even though they are responsible for most of the key public services involved in the response to COVID-19: e.g., in Italy the healthcare system is managed at the regional level, and also several containment measures (such as school foreclosures) have to be implemented by the regional government. Even more extreme is the lack of consideration for international institutions (e.g., the EU and the WHO), indicated as crucial for the management of the pandemic by only 0.23% of respondents.

**Figure 1:**
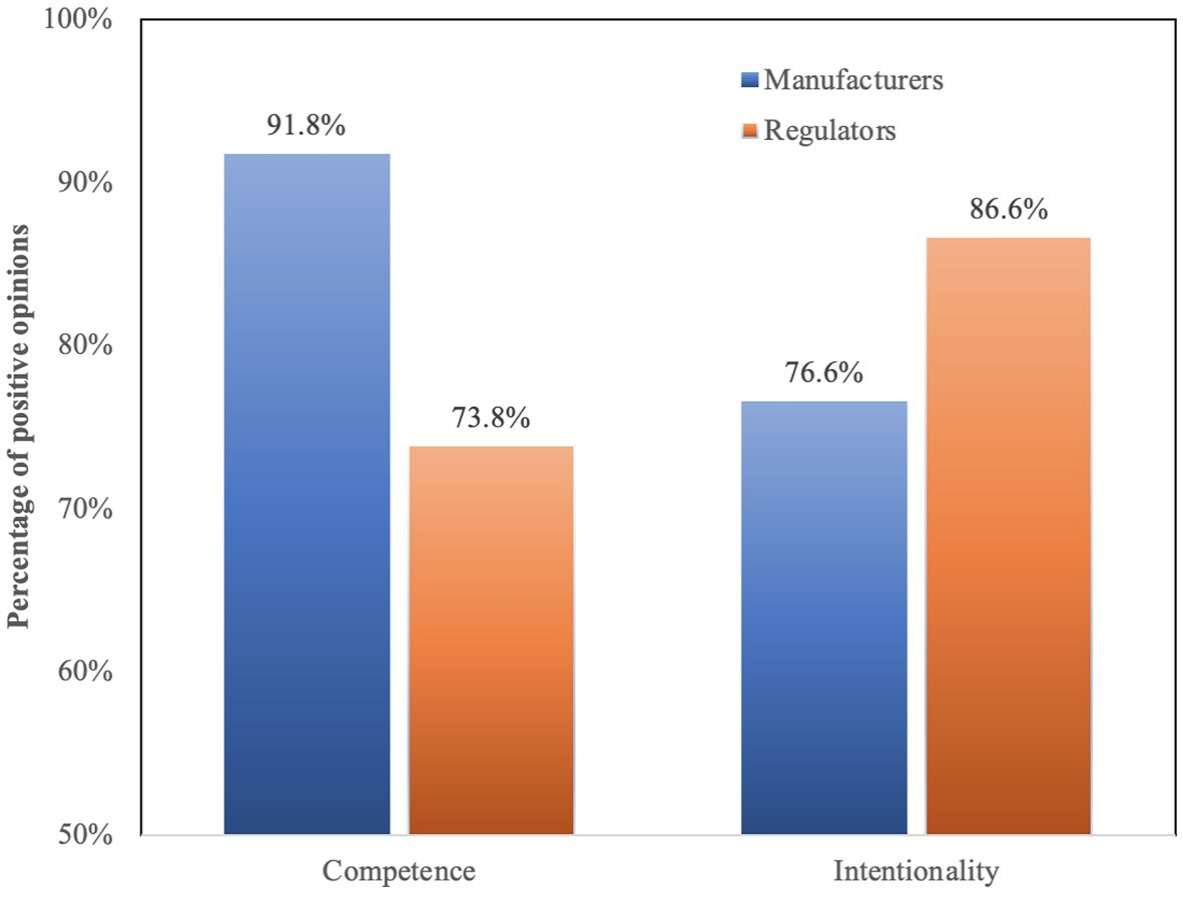
Beliefs on competence and intentionality of manufacturers and regulators regarding COVID-19 vaccines.

Moving from the overall management of the pandemic to the specific topic of COVID-19 vaccines, trust remains very high, both in *regulators* (public institutions and drug safety authorities; 77.5%) and *manufacturers* (83.5%)—AoI 2. However, as Fig. 1 shows, the main motivation behind this trust differs across these two categories: whereas regulators are trusted more for their intentions (86.6%) than for their competence (73.8%), the opposite is true for manufacturers (trust in competence = 91.8%, trust in intentionality = 76.6%); a 2 × 2 ANOVA within-s confirms the significance of this interaction [F(1, 4072) = 2307.59, p < 0.001, η_p_^2^ = 0.362]. These differences in the factors determining trust towards, respectively, regulators and manufacturers are confirmed by other results: 49.8% of respondents is concerned that the financial interests involved in the development and selling of COVID-19 vaccines may lead manufacturers to endorse practices that are not in the best interest of public health; while this worry does not undermine the overall trust towards manufacturers and vaccines, it does attest the existence of reasonable concerns in relation to the motivations of pharmaceutical companies—for instance, 20.4% of respondents consider plausible that manufacturers may exert undue influence on public authorities, whereas 31% fear that public authorities may rely too blindly on the competence of manufacturers.

### Analysis of variance and structural equation modelling

**Figure 2:**
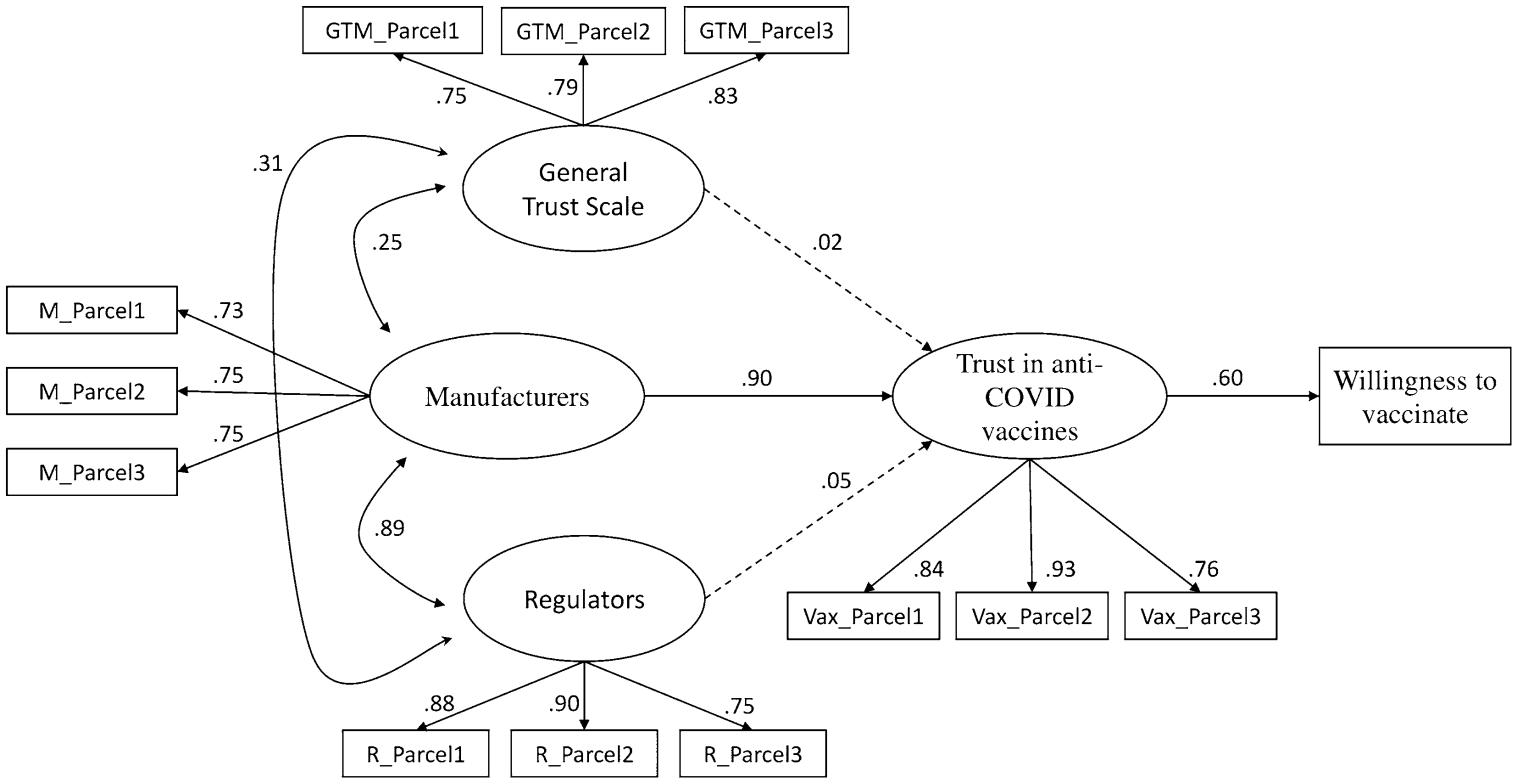
Dynamics of trust on manufacturers, regulators, and peers and their impact on confidence in vaccines and vaccination intention. Fit indexes: χ^2^ test of model fit = 1553.43; df = 59 (*p* < 0.001); Comparative Fit Index (CFI) = 0.946; Root Mean Square Error of Approximation (RMSEA) = 0.079 (90% CI 0.075–0.082); Standardized Root Mean squared Residual (SRMR) = 0.040). Scaling Correction Factor for MLR = 1.14. Parameters estimates are standardized. Dotted lines represent not significant relationships. Continuous lines represent paths with *p* < 0.001.

Crucially, these different conceptualizations have an impact on how trust in public actors modulates the intention to vaccinate against COVID-19. Crucially, these different conceptualizations have an impact on how trust in public actors modulates the intention to vaccinate against COVID-19. Using Structural Equation Modeling (SEM), we confirmed that a multivariate regression model putting in relation trust in, respectively, manufacturers, regulators, peers (i.e., as exogeneous variables), and vaccines (i.e., as the model-dependent variable) can accurately predict intention to vaccinate: more importantly, the model shows that, although trust in regulators and trust in manufacturers strongly correlate with each other (*r* = 0.89 S.E. = 0.01, _95%_CI 0.88–0.91, *p* < 0.001), see AoI 2, only the latter is crucial in generating trust in vaccines (*β* = 0.90 S.E. = 0.06, _95%_CI 0.79–1.00, *p* < 0.001). This highlights a very specific role for regulators in the trust pipeline: their function in promoting vaccination is expressed through their role as impartial evaluators of pharmaceutical products, thus improving trust in manufacturers, which in turn boosts trust in vaccines that positively predicted intention to vaccinate (*β* = 0.60 S.E. = 0.01, _95%_CI 0.57–0.63, *p* < 0.001; see Fig. 2). Not surprisingly, the only significant indirect effect on the willingness to vaccinate was that of trust in manufacturers, *αβ* = 0.54 S.E. = 0.04, _95%BC_CI 0.47–0.62, *p* < 0.001.

This result should be interpreted within the broader picture of vaccination beliefs painted by our data. Overall, positive attitudes towards vaccines were widespread, and not only with respect to COVID-19: support for curative drugs and well-tested vaccines (anti-poliomyelitis) was nearly unanimous (98.9% and 97.4%, respectively), and 85.7% of respondents endorsed the belief that the flu vaccine saves many lives every year.

Confidence in the safety of vaccines, albeit quite high (89.4%), is lower than trust in their effectiveness (93.7%): the fact that overall trust in vaccines (92.9%) mirrors closely the latter suggests that safety concerns, albeit present in the population (e.g., 36% of respondents believe that side effects of vaccines are difficult to foresee), do not undermine the overall trust in vaccination. In parallel, a shift in the nature of vaccine hesitancy is observed: besides being limited to a minority of the sample (9.3%), this attitude no longer manifests as radical skepticism on the rationale of vaccination, but rather as focused doubts on the quality of COVID-19 vaccines and suspicions on the ulterior motives of the relevant stakeholders. This is confirmed by widespread rejection of items suggesting that the political governance of the pandemic has been exaggerated (endorsed only by 5.2% of respondents), that the COVID-19 is just another type of flu (8.4%), and that it is less dangerous than other illnesses (10.7%). In contrast, vaccines are indicated as the crucial tool to counteract the pandemic by 90.5% of respondents, and 87.5% also believe their benefits to far outweigh their risks (even though as many as 16.2% consider such risks to be a concrete possibility).

As a consequence of this positive framing of vaccination, *intention to vaccinate* is very high: 88.8% of respondents either already had the vaccine or intended to get it as soon as it was made available to them; 1.2% declared health reasons preventing them from being vaccinated, while the remaining 10% showed various forms of hesitancy—namely, 2.1% did not intend to get vaccinated at all, 2.5% would do it only after being sure that it is really effective, 5.2% only when there will be guarantees of no long-term side effects, and 0.2% believed that enough other people will get vaccinated, thus making it unnecessary for them to get their shot.

Further analyses reveal two main factors affecting the intention to vaccinate: information dynamics and motivational framing.

Looking at the former, the vast majority of respondents (74.2%) considered themselves *adequately informed about vaccines*. The *information channels most frequently used* to collect data on vaccines and the pandemic are traditional media (70.3%), social media (45.5%), family physicians (29.6%), and personal acquaintances (21.9%). In terms of *reliability of information sources*, regardless of the channels used to access these sources, the subjects expressed high confidence in scientific experts (94.6%), family physicians (89.7%), and government authorities (83.7%), whereas media commentators and journalists were considered much less reliable (23.7%), at the same level of personal acquaintances (25.2%). It is worth noting that family physicians, while regarded as authoritative, appear to be rarely consulted as information sources by respondents; conversely, the severe credibility crisis of traditional journalism does not prevent users from relying on traditional media channels to gather information, possibly as an easy way to access the opinions of more trusted sources (e.g., scientists, health practitioners, politicians).

**Figure 3:**
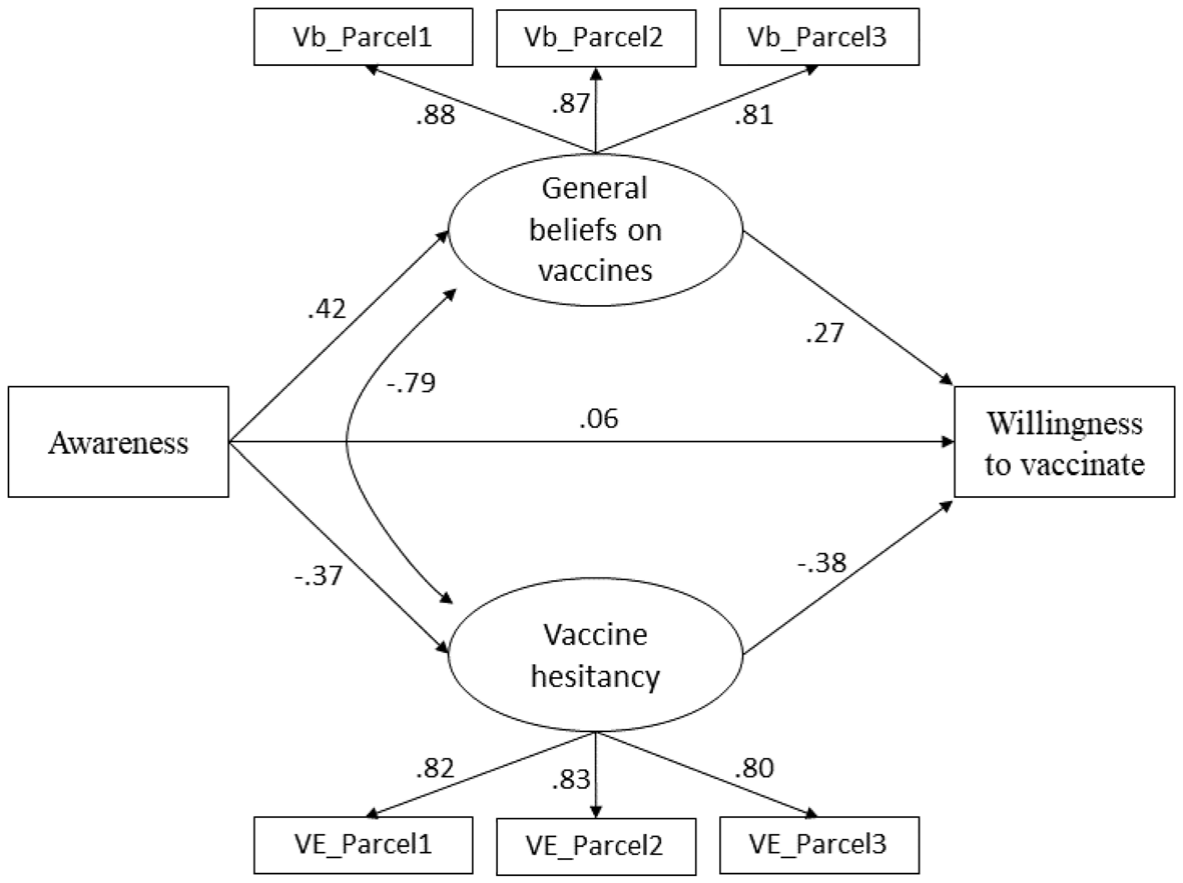
The role of considering oneself well-informed (awareness) on intention to vaccinate and its mediating factors. Fit indexes: χ^2^ test of model fit = 92.22; df = 16 (*p* < 0.001); Comparative Fit Index (CFI) = 0.991; Root Mean Square Error of Approximation (RMSEA) = 0.036 (90% CI 0.029–0.044); Standardized Root Mean squared Residual (SRMR) = 0.014). Scaling Correction Factor for MLR = 1.63. All other conventions as in Fig. 2.

More importantly, a double parallel mediation SEM model confirms the existence of several significant causal paths from information dynamics to intention to vaccinate. A first model shows (Fig. 3) that considering yourself well informed (from now on, awareness) is per se a predictor of willingness to vaccinate against COVID-19 (*β* = 0.06 S.E. = 0.02, _95%_CI 0.02–0.10, *p* = 0.001), yet this effect is strongly mediated by beliefs on the utility of vaccination (*β* = 0.27 S.E. = 0.04, _95%_CI 0.18–0.35, *p* < 0.001) and vaccine hesitancy (*β* = – 0.38 S.E. = 0.04, _95%_CI – 0.47 to – 0.29, *p* < 0.001), unsurprisingly, in opposite directions (AoI 3). Overall, the total indirect effect of the awareness on willingness to vaccinate is positive: *αβ* = 0.26 S.E. = 0.01, _95%BC_CI 0.23–0.28, *p* < 0.001.

**Figure 4:**
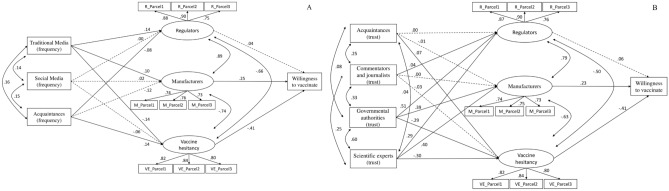
The impact of media channels (**A**) and trust in information sources (**B**) on willingness to vaccinate. (**A**) Fit indexes: χ^2^ test of model fit = 723.70; df = 51 (p < 0.001); Comparative Fit Index (CFI) = 0.966; Root Mean Square Error of Approximation (RMSEA) = 0.057 (90% CI 0.053–0.060); Standardized Root Mean squared Residual (SRMR) = 0.026). Scaling Correction Factor for MLR = 1.16. (**B**) Fit indexes: χ^2^ test of model fit = 769.81; df = 58 (*p* < 0.001); Comparative Fit Index (CFI) = 0.968; Root Mean Square Error of Approximation (RMSEA) = 0.055 (90% CI 0.051–0.058); Standardized Root Mean squared Residual (SRMR) = 0.025). Scaling Correction Factor for MLR = 1.16. All other conventions as in Fig. 2.

Two further triple parallel mediation SEM models demonstrate that both what channels are used most frequently, and what sources are considered more trustworthy, contribute to determine intention to vaccinate (AoI 4): in this case the mediating factors are trust in manufacturers, trust in regulators, and vaccine hesitancy. With respect to usage of information channels (Fig. 4A), gathering information on traditional media impacts positively on trust in both manufacturers (*β* = 0.10 S.E. = 0.02, _95%_CI 0.06–0.13, *p* < 0.001) and regulators (*β* = 0.14 S.E. = 0.02, _95%_CI 0.10–0.17, *p* < 0.001), whereas it reduces vaccine hesitancy (*β* = – 0.14 S.E. = 0.02, _95%_CI – 0.18 to – 0.11, *p* < 0.001); therefore, since intention to vaccinate is boosted by trust in manufactures (*β* = 0.25 S.E. = 0.06, _95%_CI 0.13–0.36, *p* < 0.001) and undermined by vaccine hesitancy (*β* = – 0.41 S.E. = 0.03, _95%_CI – 0.48 to – 0.35, *p* < 0.001), frequent use of traditional media results in a significant increase in willingness to vaccinate (indirect effect: α*β* = 0.09 S.E. = 0.01, _95%BC_CI 0.06–0.11, *p* < 0.001), whereas relying on personal acquaintances for collecting information has the opposite effect (α*β* = – 0.10 S.E. = 0.01, _95%BC_CI – 0.11 to – 0.06, *p* < 0.001).

As for trust in different information sources (Fig. 4B), our model confirms that only trust in scientific experts (*β* = 0.40 S.E. = 0.01, _95%_CI 0.36–0.44, *p* < 0.001) and governmental authorities (*β* = 0.39 S.E. = 0.01, _95%_CI 0.35–0.43, *p* < 0.001) simultaneously affects trust in manufacturers (positively) and vaccine hesitancy (negatively; *β* = − 0.30 S.E. = 0.02, _95%_CI − 0.34 to − 0.26, *p* < 0.001; *β* = − 0.29 S.E. = 0.02, _95%_CI − 0.33 to − 0.25, *p* < 0.001), as well as boosting also trust in regulators (*β* = 0.29 S.E. = 0.01, _95%_CI 0.26–0.32, *p* < 0.001; *β* = 0.51 S.E. = 0.01, _95%_CI 0.48–0.54, *p* < 0.001), thereby strongly supporting intention to vaccinate; in contrast, trust in media commentators and journalist is much less relevant, since it strengthens only trust in regulators (*β* = 0.04 S.E. = 0.01, _95%_CI 0.01–0.06, *p* = 0.006), which in turn does not directly predict intention to vaccinate, whereas trust in personal acquaintances is actually damaging – it cements vaccine hesitancy (*β* = 0.07 S.E. = 0.01, _95%_CI 0.03–0.10, *p* < 0.001), therefore undermining willingness to vaccinate. Moreover, it should be noted that those who rely on governmental (*αβ* = 0.24 S.E. = 0.01, _95%BC_CI 0.21–0.27, *p* < 0.001) and scientific authorities (*αβ* = 0.23 S.E. = 0.01, _95%BC_CI 0.21–0.26, *p* < 0.001) tend to be more inclined to vaccinate, contrarily to those who rely on their personal acquaintances (*αβ* = –0.03 S.E. = 0.01, _95%BC_CI – 0.05 to – 0.01, *p* = 0.001).

The other crucial predictor of intention to vaccinate is the *type of motivation* considered relevant for that behaviour. Overall, vaccination seems to be predominantly understood as *an act of responsibility to protect collective health*: only a minority of respondents (21.8%) indicates concern for their own health as the main reason to vaccinate, while the claim that getting vaccinated serves to protect others is endorsed by 91.6% of the sample (not only by preventing infection, but also by avoiding to clog up hospitals and in particular intensive care). This is mirrored in the intentions attributed to others: only 26.2% of respondents think that other people vaccinate out of self-protection, whereas 63.5% believes that others endorsing vaccination are intent on protecting public health. Interestingly, what motivation one endorses has important implications on their intention to vaccinate: regression analysis (F(2, 4042) = 1587.92, p < 0.001, R^2^ = 0.44) reveals that willingness to vaccinate is strongly predicted by the goal of protecting collective health (*b* = 0.29 S.E. = 0.005, *p* < 0.001), whereas a concern for one’s self-protection does not make respondents substantially more eager to take the vaccine (*b* = − 0.01 S.E. = 0.004, *p* < 0.001).

Another suggestive finding concerns the possibility of making COVID-19 vaccines mandatory: in spite of widespread confidence in the fact that vaccination can take us out of the pandemic (90.5% of respondents) and an overall positive attitude towards vaccination, the percentage of participants in favor of *mandatory vaccination* is much lower (59.8%) and it correlates positively with the belief that COVID-19 vaccines entail no health risks for those vaccinated (61.5%). This shows that the right to self-determination is given proper consideration, even in contexts, such as the pandemic, where orchestrated and coherent collective behaviors are essential to public health^19^. This consideration, however, does not necessarily extend to specific sectors of the population, e.g., healthcare personnel, which are considered to have a primary duty to preserve the health of others: thus, the percentage of respondents endorsing mandatory COVID-19 vaccination rises to 86.1%, with respect to health professionals (indeed, on April 1 mandatory vaccination against COVID for healthcare workers was introduced in Italy^20^).

Finally, some interesting effects of sample composition were observed (AoI 6). While trust in vaccines and intention to vaccinate remained high regardless of gender, age, socio-economic status, region of provenance, and other socio-demographic variables, two factors showed some impact on participants’ responses: *educational background* and *financial consequences* of the pandemic. However, it should be kept in mind that these results are specifically referred to our sample and its representative is not fully guaranteed.

On average, the level of instruction was rather high (74.2% of respondents were graduate or postgraduate), although a significant portion of the sample had obtained only a high school diploma or a lower title (N = 1035). More importantly, a series of one-way ANOVA between-s revealed that the level of instruction was systematically associated with a variety of indicators of positive framing of vaccines (*p*_s_ < 0.001 in all cases; see also Table 2): according to our sample, more educated respondents had more trust in COVID-19 vaccines (η_p_^2^ = 0.015), their manufacturers (η_p_^2^ = 0.01), relevant regulators (η_p_^2^ = 0.027) and institutional sources of information (η_p_^2^ = 0.012); they also tended to see themselves as better informed (η_p_^2^ = 0.017), to consider vaccines in general as beneficial (η_p_^2^ = 0.038), and to perceive the pandemic as a very severe problem (η_p_^2^ = 0.014); coherently, they exhibited lower vaccine hesitancy (η_p_^2^ = 0.011) and were less likely to associate self-serving motives to vaccination, either for themselves (η_p_^2^ = 0.026) or for others (η_p_^2^ = 0.013).

**Table 2:** ANOVA.

Independent variable	Dependent variable	F	η_p_^2^	Low		Med		High	
				M	SD	M	SD	M	SD
Educational background	Trust in COVID-19 vaccines	F(2, 4014) = 31.21	0.015	3.75	0.75	3.86	0.73	3.98	0.07
	Manufacturers	F(2, 4014) = 20.04	0.010	3.68	0.81	3.77	0.74	3.87	0.71
	Regulators	F(2, 4014) = 56.81	0.027	3.49	0.88	3.67	0.84	3.84	0.77
	Institutional Sources of information	F(2, 4014) = 24.80	0.012	3.49	0.78	3.58	0.70	3.69	0.69
	Consciousness	F(2, 4014) = 31.09	0.017	3.52	1.10	3.72	1.01	3.87	1.01
	General beliefs on vaccines	F(2, 4014) = 79.73	0.038	4.11	0.60	4.26	0.59	4.40	0.52
	General beliefs on the pandemic	F(2, 4014) = 29.91	0.015	4.23	0.88	4.40	0.77	4.47	0.73
	Vaccine hesitancy	F(2, 4014) = 23.58	0.012	1.62	0.75	1.53	0.75	1.42	0.66
	Vaccination selfish purposes (self)	F(2, 4014) = 54.73	0.027	2.50	1.45	2.12	1.29	1.96	1.20
	Vaccination selfish purposes (other)	F(2, 4014) = 28.36	0.014	3.01	1.09	2.82	1.02	2.70	1.00
Financial consequence	Trust in COVID-19 vaccines	F(2, 4014) = 21.67	0.011	3.73	0.81	3.90	0.70	4.01	0.72
	Regulators	F(2, 4014) = 30.32	0.015	3.48	0.94	3.73	0.80	3.80	0.85
	Institutional Sources of information	F(2, 4014) = 21.05	0.010	3.45	0.79	3.63	0.70	3.66	0.75
	General beliefs on vaccines	F(2, 4014) = 30.44	0.015	4.13	0.66	4.31	0.55	4.37	0.56
Economic status	Trust in COVID-19 vaccines	F(2, 3628) = 44.12	0.023	3.72	0.80	3.87	0.72	4.03	0.64
	Manufacturers	F(2, 3628) = 31.38	0.017	3.64	0.82	3.78	0.73	3.91	0.68
	Regulators	F(2, 3628) = 78.55	0.040	3.43	0.91	3.69	0.81	3.91	0.75
	Institutional Sources of information	F(2, 3628) = 31.08	0.016	3.45	0.78	3.62	0.71	3.71	0.67
	Consciousness	F(2, 3628) = 20.97	0.013	3.58	1.09	3.71	1.01	3.90	0.99
	General beliefs on vaccines	F(2, 3628) = 98.38	0.051	4.06	0.70	4.29	0.55	4.43	0.48
	General beliefs on the pandemic	F(2, 3628) = 35.03	0.018	4.22	0.90	4.38	0.78	4.52	0.68
	Mandatory vaccination	F(2, 3628) = 18.20	0.009	3.72	1.27	3.94	1.10	4.04	1.04
	Vaccine hesitancy	F(2, 3628) = 50.64	0.027	1.70	0.87	1.51	0.70	1.37	0.60
	Vaccination selfish purposes (self)	F(2, 3628) = 45.22	0.024	2.50	1.42	2.15	1.32	1.92	1.21
	Vaccination selfish purposes (other)	F(2, 3628) = 15.74	0.009	2.98	1.08	2.82	1.02	2.71	1.02

This table reported main ANOVA results. All reported results had a p < 0.001 and statistical power was always (1 − β) > 0.99.

Socio-economic status and the financial impact of the pandemic also show similar effects on respondents’ attitudes (one-way ANOVA within-s, p < 0.001 in all cases; see Table 2). Those who reported a worsening of their economic status as a result of the pandemic (19.7% of respondents) were less trustful towards COVID-19 vaccines (η_p_^2^ = 0.011), regulators (η_p_^2^ = 0.015), and institutional information sources (η_p_^2^ = 0.011), as well as expressing less confidence in vaccines in general (η_p_^2^ = 0.015), in comparison with participants whose financial situation remained stable or even improved. This seems to reflect a more basic connection between economic status and vaccination beliefs (p < 0.001 in all cases; see Table 2): higher revenues correlate positively with trust in COVID-19 vaccines (η_p_^2^ = 0.023), manufacturers (η_p_^2^ = 0.017), regulators (η_p_^2^ = 0.04), and institutional information sources (η_p_^2^ = 0.016), as well as with considering themselves well informed (η_p_^2^ = 0.013), having confidence in vaccines in general (η_p_^2^ = 0.051), taking seriously the pandemic (η_p_^2^ = 0.018), and supporting mandatory vaccination for all (η_p_^2^ = 0.009); personal wealth instead correlated negatively with vaccine hesitancy (η_p_^2^ = 0.027), considering self-protection as the main reason to vaccinate themselves (η_p_^2^ = 0.024), and attributing to others the same self-serving reasons (η_p_^2^ = 0.009).

The relevant results of the ANOVA are listed in Table 2.

## Discussion

The high levels of institutional trust observed in this study are rather unusual in Italy, and they were certainly not apparent before the onset of the pandemic: surveys conducted in recent years^11,12^ typically reported trust in public institutions in only 15–25% of respondents. In our study (N = 4096), as compared to such previous similar studies, a much larger segment of our samples population now is willing to rely on public authorities, even after more than a year of global crisis and although the rates of contagion and mortality were, at the time of the survey, still relatively high in Italy—AoI 1. Our data suggested that it is a powerful indicator of the special role played by the pandemic in shaping the relationship between institutions and citizens on this topic. Previous work, conducted in the early months of 2020^14^, interpreted the positive impact of Covid-19 on institutional trust in terms of motivated reasoning^21,22^: faced with an unprecedented global crisis, citizens quickly realized that adequate management of the situation by the competent authorities represented the only viable solution to their plight, and thus persuaded themselves to open a “line of credit” in their trust towards such authorities, in spite of whatever pre-existing reservations they may have harbored. The persistence of high levels of institutional trust in Italy after a year since the beginning of this crisis indicates that public authorities managed to live up to the citizens’ expectations, at least to some extent.

Nonetheless, a downward tendency was also noted for institutional trust, from March 2020 to April 2021, suggesting that citizens, after one year of daily co-existence with the pandemic and the resulting containment measures, have developed some reservations on how institutions have managed the crisis. These trends are in line with survey findings all across Europe^23^, and they are hardly surprising; in fact, it is actually reassuring, suggesting that the social capital built during these months is not the byproduct of a collective lack of critical scrutiny towards institutional decision making.

An important original result of this study was the identification of the different factors affecting, respectively, trust in manufacturers and trust in regulators, as well as their distinct role in determining intention to vaccinate (AoI 2). These findings describe well the different roles assigned by citizens to manufacturers and regulators in vaccine development: whereas the former are expected to demonstrate technical prowess in delivering effective and safe vaccines (competence), the latter are valued mostly for their independence from private interest and their dedication to public health (intentionality). Moreover, while trust in regulators correlates strongly with trust in manufacturers, only the latter is a relevant predictor of intention to vaccinate. This has important policy implications: in particular, regulators should resist the temptation of building their own social capital at the expense of the reputation of manufacturers, since this may in turn trigger a crisis of confidence in vaccines; at the same time, manufacturers should appreciate that their own PR blunders and public contradictions may not be reparable by regulators, no matter how hard the latter try (both factors, arguably, played a part in the AstraZeneca communication disaster^15^).

Looking at background beliefs against which these trust dynamics were observed, two main results stand out: (i) a largely positive outlook on vaccines in general, as well as (ii) some nuanced forms of vaccine hesitancy in a minority of respondents (AoI 3). The former result is consistent with other observations of a COVID-induced increase in overall trust towards medical science^24,25^; of particular note is the strong endorsement of flu vaccines observed in our sample, which is in sharp contrast with the low rates of influenza vaccination in Italy before the pandemic, even among healthcare workers^26^, and the characteristic under-appreciation of the dangers linked to the flu manifested in this country (e.g., in March 2019 only 15% of Italian respondents believed that people still die of the flu in the EU^27^). As for vaccine hesitancy, we documented it only in a small minority of our sample: in general, it did not seriously undermine confidence in the effectiveness of vaccines, whereas some relatively specific (and not always entirely unreasonable) doubts were focused on their safety and on possible conflicts of interest for some key stakeholders (for similar patterns in recent vaccine hesitancy towards flu vaccines, see^26^). Taken together, our results seem to suggest that vaccine hesitancy is best understood as a wide spectrum of reservations, deeply affected by contextual factors, and only rarely manifesting as blanket refusal of vaccination, consistently with recent meta-analysis of the phenomenon^28,29^.

Given the overall positive mindset of participants towards vaccines and public institutions, also their self-reported intention to vaccinate was remarkably high. More interestingly, our results shed some light on what factors, either epistemic or motivational, are most relevant in determining willingness to vaccinate (AoI 5), often via the crucial mediation of trust in manufacturers.

Regarding the role of knowledge, we collected evidence of how information dynamics modulate intention to vaccinate (AoI 4), over and above any positive effect of societal-level trust in science^30^: collecting information on the pandemic and COVID-19 vaccines provides good reasons to endorse vaccination, yet this effect depends on the specifics of the information gathering process^31^; those who consult traditional media and trust governmental and scientific authorities are much more willing to vaccinate than those who rely on their personal acquaintances for health-related information, whereas frequenting social media does no harm and listening to journalists does no good (for similar findings in the UK and Ireland, see^32^). Some of these results are consistent with other observation in the recent literature: feeling knowledgeable on the pandemic increased willingness to vaccinate against COVID-19 in a US sample^33^, whereas a positive effect of trust in scientific experts and governmental institutions on vaccination intention was also observed with respect to anti-H1N1 vaccination in France^34^. Other findings were less predictable: remarkably, in our data frequent use of social media has neither positive nor negative consequences on intention to vaccinate (in contrast with data from a US sample^33^). This reminds us that at least some concerns on these platforms are misplaced: the problem does not appear to be intrinsic to the technology, but rather dependent on what sources are most heeded by users^35,36^.

As for motivational factors, our results suggest that the specific goal participants have in mind when considering vaccination has an impact on their intention to get vaccinated. In particular, we observed that (i) the large majority of respondents see vaccination as an act of social responsibility, i.e. something to be done to benefit all rather than just ourselves, and (ii) the same collectivist motivation is overwhelmingly attributed to others; even more importantly, (iii) framing vaccination in these terms is a powerful predictor of vaccination intention, whereas a self-protective frame of mind does not increase substantially willingness to vaccinate against COVID-19. Rather that signaling overwhelming altruism in the population, these data suggest that people are acutely aware of the interdependencies created by the pandemic: participants are mindful that the achievement of their personal goals is predicated, now more than ever, on the choices made by other society members. To put it simply, self-protection is seen as unattainable without adequate coordination at the society level. This result is consistent with previous game theoretical models of intention to vaccinate against influenza^37^ and may have far-reaching implications on message crafting in vaccination campaigns, since it suggests that emphasizing different reasons may produce vast differences in outcomes.

The last relevant set of findings concern two socio-demographic factors that were found to modulate participants’ attitudes towards vaccines: educational level and socio-economic status, especially in relation to the pandemic. Although none of these factors were strong enough to subvert the overall trust in vaccines and drastically undermine intention to vaccinate, they did have a systematic impact on people’s beliefs on the pandemic and its management.

Regarding educational background, several small but convergent effects show that vaccines are perceived more positively by people with higher instruction levels (AoI 6). This is a recurrent finding in the literature: in recent studies, the same pattern has been observed in France^13^, the UK^38^, and the US^33,39^.

Similarly, people with lower socio-economic status or who suffered more financially for the pandemic reported lower trust in regulators, institutional information sources, and vaccines—both in general and towards those specifically targeting COVID-19 (for similar results in other countries, see^13,38–40^). Taken together, these data reveal a systematic connection between economic status and vaccination attitudes: more affluent people have a more positive mindset regarding vaccination—a fact mirrored by the lower rates of vaccine hesitancy typically observed in richer countries.

While it is important to keep in mind that these effects only temper the intention to vaccinate in our sample, without subverting it, the paradoxical nature of these findings deserves emphasis: poorer and less instructed people are the most severely affected by the pandemic, both individually^41–43^ and at the country level^44,45^, and thus they stand to gain the most by prompt universal adoption of COVID-19 vaccination. It is therefore ironic and worrying to observe that precisely these categories are those most likely to exhibit some form of vaccine hesitancy: policy makers would do well to heed these cautionary signs and devise strategies to overcome skepticism and resistance in the weakest segments of the population.

## Limitations

In considering the findings of this study, it is important to also take into account the limitations of this research.

First of all the sampling procedure: in this survey, we made use of the snowball sampling procedure that is known to be partially biased. For example, it is not a completely random sampling approach since the final sample mainly depends on both experimenters’ contacts and populations networks. However, to attempt to lessen this issue, we carefully shared our questionnaire using different social media platforms (to collect data from all age groups) and differentiated thematic groups (to balance socio economic status). Moreover, by using several different social media groups we reached a very large and differentiated population, as highlighted by demographics statistic.

However, we would specify that our results are specifically referred to our sampled population. Even though all the experimenters involved in this study did their best to ensure populations’ representativeness by sharing the questionnaire across different ages, geographical areas, and socio economic statuses, the complete representativeness of our sample was not guaranteed. Nevertheless, to increase population variability and mitigate such a problem we both collected a very large number of participants (N = 4762) and we carefully checked its adherence to the Italian population, as specified in Table 1 and reported in the text.

Secondly, as in most online surveys, it was not possible to determine the acceptance rate of our participants. Indeed, despite our sampled population seems to be comparable to the Italian one (in terms of socio demographic information), we could not control whether some specific subpopulations were reluctant to participate in our survey.

Lastly, it should be kept in mind that these results concern a specific phase of the pandemic, and any attempt to generalize our findings both to the general Italian context and to other countries should be conscientious and discussed only in a comparative perspective.

## Materials and methods

### Sample

An initial sample of N = 4762 participants was collected for the purpose of this study, between March 26 and April 7, 2021. Participants were recruited through a snowball sampling procedure. In particular, the survey was disseminated by the researchers using their networks, by means of different social media platforms, emails, and private messages (i.e., mailing lists available to authors). Recruited participants were then asked to share the questionnaire with their networks. We chose this sampling technique because our interest was to investigate the vaccines related trust among the general population, in such a specific period of the emergency.

Even though this approach is a non-random sampling where often generalization or representativeness are not sought after, we recruited a large number of participants in order to make our data as generalizable as possible and to avoid other related limitations.

After data collection 666 participants were excluded based on the following exclusion criteria:

1. 648 participants did not complete more than two sections, thus leaving the survey before its end;
2. 18 participants took less than 5 min (more than 2SD over the RTs mean) to complete the whole survey.

These exclusion criteria were established before starting data collection to avoid biasing the data sample.

All the remaining N = 4096 subjects (F = 2216) successfully completed the survey. Informed consent was collected from all participants before starting data collection; participants were informed in advance about the study and its main objective and purposes. Furthermore, we received the approval by the Ethical Commission of the National Research Council of Italy that with notification N.0053772/2021 has considered this research compliant with all current ethical and privacy guidelines for behavioural research.

The main characteristics of the sample are summarized in Table 1.

### Survey structure and design

The survey was run on Qualtrics, an IT platform designed for online questionnaires. The study consisted of a single session that lasted 15 min on average. By accessing a single un-reusable link, participants could run the survey directly from their own laptops, smartphones, or tablets. We designed a within-subjects paradigm in which participants responded to a total of 79 different items. The questionnaire was based on the socio-cognitive model of trust developed by Castelfranchi and Falcone^10^ and explored participants’ opinions on several dimensions, in order to characterize people’s attitudes towards COVID-19 vaccination in Italy. Each item was measured using a 5-point Likert scale (from “strongly disagree” to “strongly agree” for most of the items). Subjects firstly completed the Yamagishi General Trust Scale^46^, a 5-item questionnaire aggregated to measure an individual’s general level of trust toward other people (α = 0.82). Then, they indicated which public authority was the most appropriate to make decisions in relation to the pandemic crisis, and expressed their general trust in public institutions for the management of the pandemic.

Successively, participants completed nine different sections in a semi-randomized order (sections 6 to 9 were shown sequentially).

- Analysis of *competence*, *intention* and overall *trustworthiness* in *vaccine manufacturers*: the purpose of this section was to investigate, by means of 5 different items, citizens’ perception of the general reliability of scientific manufacturers and their ethical willingness to safeguard public health. A composite score, named “*manufacturers*”, was created for this section using all the above mentioned items (α = 0.78).
- Analysis of *competence*, *intention* and overall *trustworthiness* in the *regulators* responsible for ensuring the safety and efficacy of the vaccines: the purpose of this section was to investigate citizens’ perception of the general reliability of *regulators* (both political and healthcare) and their ethical willingness to safeguard public health. A composite score, named “*regulators*”, was made by averaging the 5 items of this section (α = 0.86).
- A further section was meant to explore the subjects’ perspective on *vaccines and drugs in general*, with a specific focus on their safety, efficacy and overall trustworthiness. The 7 items that were found to best characterize the participants’ “*general beliefs on vaccines*” were aggregated in a single factor (α = 0.86). The remaining 3 items, concerning non-vaccine drugs, were excluded from the analysis.
- Section 5 focused on both *frequency of use* of various information channels and *trust* in several information sources. Additionally, subjects reported how informed they considered themselves to be (*awareness*) concerning COVID-19 vaccines.
- The subsequent section included 4 items describing subjects’ “vaccine hesitancy”. Specifically, participants expressed their degree of accordance with various anti-vax beliefs. The 4 items were aggregated in a single factor (α = 0.86).
- Section 7 focused on trust in COVID-19 vaccines: it included 3 items describing “general beliefs on the pandemic” (α = 0.78), while 3 other items concerned the effects of COVID-19 vaccines on personal health.
- A single item was used to register subjects’ “willingness to vaccinate”. Participants could choose among 7 response options, that were coded as follows: the first 2 options (“I have already been vaccinated” and “I will get the vaccine as soon as it’s my turn”) indicated strong willingness to vaccinate; the following 2 options (“I will take the vaccine only when I will be sure that there are no side effects” and “I will take the vaccine only when I will be sure that it is truly effective”) were interpreted as temporary indecisiveness; finally, options like “I have no intention of getting vaccinated” and “It is not necessary for me to get vaccinated” showed a general hostility to vaccination; the last option, “In my case, the vaccine is definitely not recommended for medical reasons”, was included to avoid misinterpreting as vaccine hesitancy what is simply the consequence of specific health conditions (participants who opted for this answer were not considered as either willing or unwilling to vaccinate).
- Section 9 started with 2 items on people’s views on “*mandatory vaccination*” against COVID (both in general and related to healthcare workers); then it included 8 items exploring the participants’ motivational framework concerning COVID-19 vaccination (collectivist vs individualist).
- Lastly, we measured trust towards each of the 4 available vaccines in Europe at the time of data collection. These items, together with two previous items from section 7, were grouped together in the factor labelled “*trust in* COVID-19 *vaccines*” (α = 0.88). An additional question probed for changes in public opinion towards COVID-19 vaccines, as the result of the decision of some European governments to interrupt the administration of the AstraZeneca vaccine in April 2021.

Finally, subjects provided some basic socio-demographic information, reporting also how strongly they had been affected by the pandemic (e.g., the financial backlash of the crisis and the severity of the symptoms manifested among their personal acquaintances). Table 3 summarizes the principal factors considered.

**Table 3:** Principal factors.

Factors	Items	α
General trust scale	Section 1(1–6)	0.82
Manufacturers	Section 2 (1–5)	0.78
Regulators	Section 3 (1–5)	0.86
General beliefs on vaccines	Section 4 (1, 3, 4, 7–10)	0.86
Awareness	Section 5 (1)	NA
Vaccine hesitancy	Section 6 (1–4)	0.86
General beliefs on the pandemic	Section 7 (1–3)	0.78
Willingness to vaccinate	Section 8 (1)	NA
Mandatory vaccination	Section 9 (1,2)	0.79
Trust in COVID-19 vaccines	Section 7 (5,6), Section 10 (1–4)	0.88

### Statistical analysis

IBM SPSS 26^47^ was used for all statistical analysis. The structural equation models were built and run on Mplus 8.5^48^, using Maximum Likelihood estimation with Robust standard errors, in order to be conservative regarding the non-normality of the variables’ distribution^49^. An item parceling procedure^50^ was employed to assess the measurement indicators for all the latent variables of the models. Item parceling procedure combines the items of a given scale into a restricted set of items to diminish the dimensionality and the number of parameters estimated in the model, thus resulting in a more parsimonious measurement model and more reliable parameter estimates^51^. The item parcels were created by randomly grouping the items of each scale into three separate cluster sets (i.e., parcels) and by averaging the item scores within each cluster. All the parallel mediation models (i.e., Figs. 3, 4) were conceived through a transmittal approach^52,53^. Indirect effects (i.e., α*β*) and their standard errors were computed through a non-parametric bootstrap procedure (*N* = 5000)^48^; 95% bias-corrected bootstrap confidence intervals are provided (i.e., _95%BC_CI) as these intervals take the non-normality of the parameter estimate distribution into account^48^. The significance level of all analyses was set to *α* = 0.05. All variables were checked for normality by the Shapiro–Wilk test and for homoscedasticity by Levene test. In the case of violation of the above assumptions, data normal distribution for parametric analysis was assessed using skewness and kurtosis measures for large-sized samples^47,54^. When the distribution of the sample was normal, parametric analysis of variance was performed on the composite scores described in the previous subsection. Parametric post-hoc pairwise comparisons were corrected using Bonferroni correction and only ran following a significant main effect.

Between-s differences were assessed by means of several one- and two-way ANOVAs. The relationships between our factors and the general willingness to vaccinate (AoI 5) were investigated through SEM.
